# High Risk of Lead Contamination for Scavengers in an Area with High Moose Hunting Success

**DOI:** 10.1371/journal.pone.0111546

**Published:** 2014-11-12

**Authors:** Pierre Legagneux, Pauline Suffice, Jean-Sébastien Messier, Frédérick Lelievre, Junior A. Tremblay, Charles Maisonneuve, Richard Saint-Louis, Joël Bêty

**Affiliations:** 1 Département de biologie, chimie et géographie & Centre d'études nordiques, Université du Quebec à Rimouski, Rimouski, Quebec, Canada; 2 Ministère du Développement durable, de l'Environnement, de la Faune et des Parcs, Quebec, Quebec, Canada; 3 Sciences and Technology Branch, Environment Canada, Quebec, Quebec, Canada; USGS National Wildlife Health Center, United States of America

## Abstract

Top predators and scavengers are vulnerable to pollutants, particularly those accumulated along the food chain. Lead accumulation can induce severe disorders and alter survival both in mammals (including humans) and in birds. A potential source of lead poisoning in wild animals, and especially in scavengers, results from the consumption of ammunition residues in the tissues of big game killed by hunters. For two consecutive years we quantified the level lead exposure in individuals of a sentinel scavenger species, the common raven (*Corvus corax*), captured during the moose (*Alces alces*) hunting season in eastern Quebec, Canada. The source of the lead contamination was also determined using stable isotope analyses. Finally, we identified the different scavenger species that could potentially be exposed to lead by installing automatic cameras targeting moose gut piles. Blood lead concentration in ravens increased over time, indicating lead accumulation over the moose-hunting season. Using a contamination threshold of 100 µg.L^−1^, more than 50% of individuals were lead-contaminated during the moose hunting period. Lead concentration was twice as high in one year compared to the other, matching the number of rifle-shot moose in the area. Non-contaminated birds exhibited no ammunition isotope signatures. The isotope signature of the lead detected in contaminated ravens tended towards the signature from lead ammunition. We also found that black bears (*Ursus americanus*), golden eagles and bald eagles (*Aquila chrysaetos* and *Haliaeetus leucocephalus*, two species of conservation concern) scavenged heavily on moose viscera left by hunters. Our unequivocal results agree with other studies and further motivate the use of non-toxic ammunition for big game hunting.

## Introduction

Lead bioaccumulation is a threat to both human [1] and wildlife health [2],[3]. Lead has irreversible negative effects on general health, reproduction and behaviour and can potentially lead to death [3]. Sub-lethal toxic effects are exerted on the nervous system, kidneys and circulatory system, resulting in physiological, biochemical, immunological and behavioural (e.g., locomotor) changes [3]–[7]. Top predators and scavengers are essential in ecosystem functioning [8]–[11]. Indeed, trophic interactions via facultative scavenging can impact ecosystem stability and persistence [12], [13]. Because of their position at the top of the food chain, predators and especially scavengers are vulnerable to the bioaccumulation of pollutants [10]. Lifetime cumulative of sub-lethal lead exposures may have more dramatic consequences for wild animals than recent exposure [14].

In birds, the deleterious effects of lead were first reported in waterfowl that had ingested lead shot as part of their diet [15], [16]. Similarly to waterfowl, galliforms and doves also ingest shot pellets as grit to be retained in their gizzards [3]. Many birds of prey that rely on upland game birds and mammals as a primary food source are at risk of secondary lead poisoning from the ingestion of lead ammunition that has been consumed by their prey [17]. Lead toxicity can be severe; mortality can occur following the ingestion of just one lead shot pellet [18]. In North America, ingestion by bald (*Haliaeetus leucocephalus*) or golden eagles (*Aquila chrysaetos*) of waterfowl wounded or killed by lead ammunition was responsible for 10 to 15% of post-fledging mortalities [17], [19].

To eliminate this mode of lead exposure for migratory birds, lead shot cartridges were banned in the United States in 1991. In Canada, ban started in 1997 for hunting waterfowl and migratory birds within 200 m of water basins, and in 1999 the ban was extended to dry land (this only concerns waterfowl hunting). The enforcement of these wildlife protection laws likely also benefited predators, scavengers and humans [20]. However, lead ammunition is still in use in varmint and upland game hunting [21] as well as in big game hunting [22].

Ingestion of prey contaminated by lead shot has been historically considered the main pathway of contamination of birds of prey [23]. More recently, retention of ammunition residues in the tissues of carcasses of hunted big game was identified as another main source of contamination for wildlife [3]. When penetrating a large animal, lead bullets can fragment and scattered within a minimal radius of 20 cm from the wound [24], [25]. Many species of conservation concern scavenge on large ungulate carcasses or on viscera discarded by hunters and can be impacted by lead contamination [2], [26], [27]. Eating carcasses or gut piles with embedded lead fragments can thus be a major source of contamination for some top predator species including humans [1], [2], [28]. Our study focuses on the most abundant bird scavenger, the common raven (*Corvus corax*), as a sentinel species for contamination from lead bullet fragments. In dense forest, ravens are among the first species to detect carcasses and often scavenge in groups [29]. Ravens have been shown to be an adequate sentinel to assess potential lead exposure from big game hunting [28]. While bones and brain are the target tissues for long-term storage of lead, blood represents only 2% of total lead body burden [30]. However, blood lead levels can assess recent ingestion due to the rapid turnover and are pertinent to assessing temporal changes in lead concentration in the diet due to shifts in food intake, with a half-life of about two weeks [28], [31].

Here, our main aim was to quantify blood lead concentration in ravens during two consecutive years from individuals captured within the big game hunting season in a high moose density area in Quebec, eastern Canada. The two years greatly differed in the total of moose harvested. We hypothesized higher blood lead levels in years with more intense hunting pressure. We further discriminated the origin of lead in raven blood samples between natural sources (e.g. atmospheric deposition) and lead originating from ammunition using stable isotope analyses. Since most lead-based ammunitions sold in Canada are manufactured in the USA, their stable lead (Pb) isotope ratio signature is similar to the lead ores extracted there [32]. Finally, we identified the different scavenger species that could be impacted by deploying automatic cameras targeting moose gut piles during the hunting season.

## Material and Methods

### Study site and species

The study was conducted in 2011 and 2012 close to Rimouski (N48°27′; W68°32′), which is located in the Bas-St-Laurent region in the province of Quebec. This region supports one of the highest moose densities in Quebec, with up to 2.5 moose/km^2^ (MDDEFP, unpubl. data). These high densities lead to an elevated hunting success, creating high numbers of moose carcasses and gut piles potentially available to scavengers. In this region (Zone 2; http://www.mddefp.gouv.qc.ca/faune/reglementation/chasse/pdf/Carte-Zone-02.pdf), a count of the number of moose rifle-shot by hunters was available for each hunting day. In 2012, hunting was only authorized for bull and calf moose in most parts of the hunting area while cow, bull and calf could be shot in 2011. Rifle hunting of other species like white-tailed deer (*Odocoileus virginianus*) was illegal or negligible compared to moose in the study region during the study period. Within the Bas-Saint Laurent region, our main study site was located in an area of 196 km^2^ managed by an outfitter (Pourvoirie Le chasseur, N48°07′; W68°00′). In this area about 100 moose are harvested annually. Within the outfitter-managed area, we deployed three gut piles with some moose provided by local hunters and captured scavenging ravens using a net launcher (Trapping Innovations, LLC). Piling guts at specific sites is a common practice in areas managed by outfitters and in some wildlife reserves in Quebec. Collecting ravens before the start and at the very beginning of the moose hunting season was virtually unfeasible at the outfitter-managed area since ravens were spread all over the territory, were hard to see in this forested area and difficult to attract to bait prior to late September. To better estimate the lead concentration of ravens prior big game hunting season at the very beginning of the hunting season, we also collected ravens using nontoxic shotgun ammunition in an area located at 70 km from the outfitter-managed area, close to Rimouski's Ecocentre (N48°24′; W68°34′). In this area, bird trapping and baiting was logistically unfeasible. Ravens are observed all year long and can be more easily collected in this agro-forested area.

### Automatic cameras

At the three capture sites (see above), automatic cameras (Reconyx PM35T25 operating 24 hours) were deployed from mid-September to mid-October each year to assess the presence of the various scavenging species. All pictures were scanned by a single observer (PL). On pictures with foraging activities, date, hour, species and maximum number of individuals sighted simultaneously were considered.

### Blood lead concentration in the common raven

Similar blood samples were taken on live and dead individuals. Two to three ml of fresh blood was taken directly from the heart of freshly collected dead birds and the same volume was collected in jugular vein of live ravens using a 25 G needle and 3 cc syringe. The collected volume was then separated into two heparinized tubes and kept frozen until analysis.

Blood lead concentration was measured using inductively coupled plasma mass spectrometry (ICP-MS) at the Institut National de Santé Publique du Québec (INSPQ) according to their standardized analytical protocol.

### Stable Isotope analyses

A subsample of 34 birds was used for stable isotopes analyses. Samples were prepared following Pariseau et al. [33]. Briefly, samples of 0.13 to 0.5 ml of lyophilized blood were acid digested in a Teflon tube with a solution of 0.6 ml of concentrated nitric acid at >69% and 0.5 ml of hydrogen peroxide at 30% for 2 hours in a 50°C water bath. The acid digest was diluted to 6 ml with ultrapure water (18 MΩ) and centrifuged at 5000 g for 10 minutes. Stable Pb isotopes (^206^Pb, ^207^Pb, and ^208^Pb) were measured by ICP-MS using an Agilent 7500c quadrupole instrument. Each solution was spiked with thallium at 20 µg/L to monitor the stability of the mass spectrometer by measuring the ratio Tl^205^/Tl^203^; the mean value was 2.365±0,005 (n = 33).

### Statistical analyses

We used Generalized Linear Models on log-transformed data (to meet assumption of normality) to test if lead concentration differed between the two years or according to date that birds were sampled. We also tested for an interaction between date and year. We compared lead stable isotope signatures using (^206^Pb/^207^Pb and ^206^Pb/^208^Pb ratios) obtained from blood with signatures available in the literature for soils and lichen in Quebec [34], [35] and for lead ammunition [17], [36]. We generated a normal distribution of soil and ammunition isotopic signatures using mean and standard deviations reported in the literature and compared it to the signatures of contaminated and uncontaminated birds using a Kolmogorov-Smirnov tests. Blood lead concentrations under 100 µg.L^−1^ were considered the baseline level of exposure from natural lead sources [28]. We analyzed the pictures taken by automated camera as follow: for each day and for each baited site, we visually search all photos and identified the different species sighted. We then compiled the frequency of occurrence for each species for the entire study period, as well as the maximum number of individuals of a species sighted simultaneously.

### Ethics statement

Licensed hunters registered at the outfitter Pourvoirie Le chasseur provided the moose viscera deployed in this study. The Animal Care Committee of Ministère du Développement durable, de l′Environnement de la Faune et des Parcs (MDDEFP) approved the different protocols used in this study (CPA Faune 2011-31 and CPA Faune 2012-29 to JAT). Raven collection was approved by the MDDEFP (Permit 20120912-011-01-S-F to JB).

## Results

**Figure 1:**
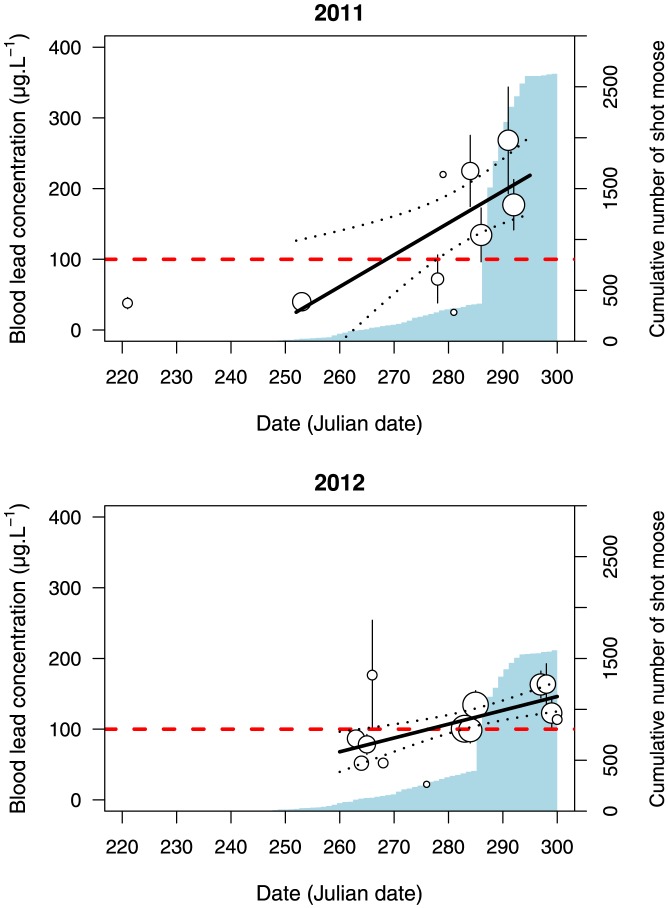
Relationship between blood lead concentrations in common ravens and date in 2011 and 2012. Dot size is proportional to the log of the sample size. Error bars represents SE. Dotted lines represent the 95%CI. The light blue barplot illustrates the cumulative number of rifle-shot moose in the hunting area selected for the study. Ravens with no sign of clinical contamination were reported to have lead concentration <100 µg.L^−1^ (horizontal red dashed line) according to [28].

During the 2011 and 2012 hunting seasons, 39 and 71 different individuals were live captured within the outfitter-managed area and sampled, respectively. In 2011 and 2012, 11 and 26 ravens were shot slightly before and during the moose-hunting season near the Rimouski's Ecocentre. Because blood lead concentration of individuals sampled during the moose hunting period at the two study areas (outfitter-managed area and Ecocentre area) were similar (F_1,111_ = 1.97; P = 0.17, after controlling for date with year consider as a random factor, test performed during the moose hunting period), we pooled all samples gathered during the hunting season for subsequent statistical analyses. During the moose hunting period, we found that blood lead concentration in the common raven increased over time in both years (F_1,137_ = 16.23; P<0.001; Fig. 1). Lead concentration also differed between years (F_1,137_ = 3.98; P = 0.048 after controlling for date; Fig.1) with a higher concentration in 2011 compared to 2012. Slope was greater in 2011 compared to 2012 (β = 3.32±1.12 s.e. in 2011 and β = 1.95±0.54 s.e. in 2012; significant interaction: F_1,143_ = 6.78; P = 0.01, analysis performed for data during the hunting period). Differences in blood lead concentration between years coincided with differences in the number of rifle-shot moose in the area, with approximately half the moose shot in 2012 than in 2011 (Fig.1). Using a contamination threshold of 100 µg.L^−1^, 63% and 51% (in 2011 and 2012 respectively) of individuals were considered lead-contaminated during the moose hunting period. When restricted to Julian days >285 (the date at which rifle hunting was allowed over the entire study region) those percentages were 82% and 60% in 2011 and 2012 respectively.

**Figure 2:**
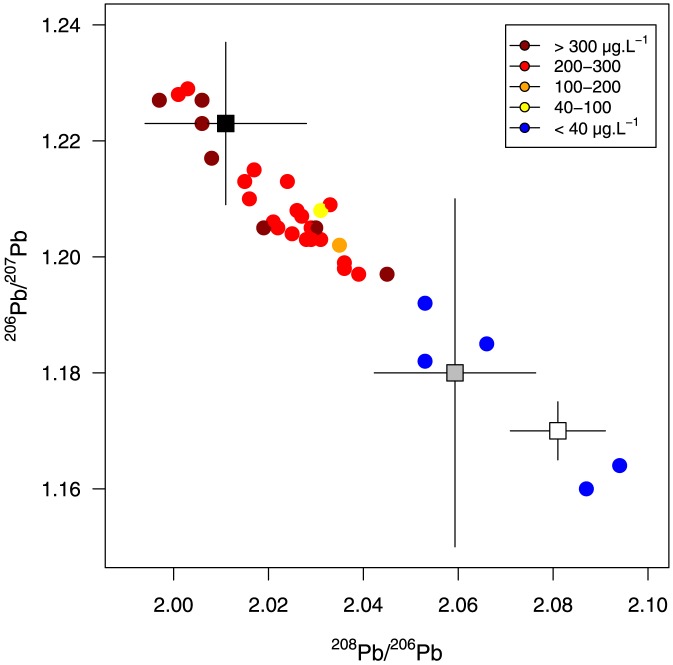
Lead stable isotope ratios measured in blood samples collected from common ravens. Data were color coded for four ranges of blood lead concentration. Suggested sources of lead using ^206^Pb/^207^Pb and ^208^Pb/^206^Pb reported in the literature for soil [34] and lichen [35] (gray and white squares respectively) and ammunition [36] (black square) are also presented. Errors bars represent the standard deviation. Dotted lines represent mean and standard errors for ^206^Pb/^207^Pb reported in [22] for ammunition. Samples collected from common ravens are presented as coloured dots (colours depend on blood lead concentration).

The isotopic signature of the lead found in the blood of non-contaminated birds (<100 µg.L^−1^) did not differ from the signature of soil or lichen lead (k-s tests: D = 0.33 and P>0.89 for both ^206^Pb/^207^Pb and ^206^Pb/^208^Pb ratios; Fig. 2) but differed from ammunition signatures (k-s tests: D = 0.86 and P<0.001 for both ^206^Pb/^207^Pb and ^206^Pb/^208^Pb ratios; Fig. 2). Signatures of lead contaminated birds (>100 µg.L^−1^) differed significantly from soil and lichen signatures (D>0.85 and P<0.0001; Fig. 2) and tended towards ammunition signatures (D = 0.36, P = 0.06 and D = 0.5, P = 0.002 for ^206^Pb/^208^Pb and ^206^Pb/^207^Pb respectively; Fig. 2). The photographs taken by the automatic cameras confirmed the common raven as the most frequent species scavenging on moose guts left by hunters. Other common scavengers identified were black bears, coyotes, golden and bald eagles (Table 1).

**Table 1:** Frequency of observations (number of days with species observed/total number of days surveyed) and maximum number of individuals sighted simultaneously for each species observed feeding on moose gut pile using Reconyx automatic cameras deployed at three baited sites in September-October 2011 and 2012.

	All (59 days)		2011 (28 days)		2012 (31 days)	
	Freq %	Max	Freq %	Max	Freq %	Max
Common raven *Corvus corax*	73	25	71	23	74	25
Black bear *Ursus americanus*	58	4	57	4	58	2
Coyote *Canis latrans*	27	1	4	1	52	1
Golden eagle *Aquila chrysaetos*	17	3	4	1	29	3
Bald eagle *Haliaeetus leucocephalus*	15	3	18	3	13	2
Gray Jay *Perisoreus canadensis*	5	1	11	1	0	0
Snowshoe hare *Lepus americanus*	2	1	4	1	0	0

Only pictures with foraging animals were kept in the analysis.

## Discussion

Since the 1960s, lead poisoning has been identified as a threat to wildlife [15], [37]. As opposed to contamination through prey exposed to lead ammunition (e.g. in prey gizzards or tissues), scavengers can also be directly contaminated from eating lead bullet fragments in gut piles discarded by big game hunters (Fisher et al. 2006). By combining data on the seasonal and annual changes in blood lead levels with stable isotopes analyses in the common raven, a sentinel scavenger, our study provides the first evidence of the risk posed by lead ammunition used for big game hunting in Canada.

Our results concur with the patterns of common raven blood lead concentration reported in Craighead and Bedrosian [28] in Wyoming; there were low levels of raven contamination outside of the big game hunting season compared to increasing levels during the hunting season. Considering a contamination threshold of 100 µg.L^−1^ [28], the high proportion of contaminated birds found in this study and the fact that moose hunting is widely distributed in the area suggest that contamination likely occurs at broad spatial scales. Moreover, ravens are able to cover great distances during the non-breeding period with home ranges >7000 km^2^ [38] increasing the opportunity to find contaminated carcasses. Stable isotopes are more and more used to characterize the origin of lead contamination n in different tissues [2], [39]. First, all birds tested with background lead levels exhibited no ammunition isotope signatures. Isotopic lead signatures of contaminated birds were constituted of a mix from natural and ammunition sources. This mix of sources could indicate that birds have been foraging on various food resources with different contamination levels. Outside the breeding season, diet of ravens is indeed variable and mostly composed of mammals, seeds, garbage and carrions [40] for which lead isotopic signatures were not assessed. Individuals with high lead blood concentrations had lead isotopic signatures close to the ones of lead ammunition reported by Tsuji et al. [36] or Church et al. [22] indicating that lead ammunition was the most likely source of contamination. Isotopic signatures of ammunition can greatly vary depending on the origin of the lead used [39]. In Quebec, most lead-based ammunitions sold are manufactured in the USA where isotopic signatures of different ammunitions are very similar to each other [22], [32], [36].

Finally, lead concentrations differed between the two years of the study, corresponding to the annual variation in moose harvest rate. In 2011, moose hunting of any sex and age was permitted and the number of moose killed (and hence gut piles discarded by hunters) was 66% higher compared to 2012 when only males and calf were hunted in most parts of our study area. Although only based on two contrasted years, this result concurs with previous studies showing that lead contamination was closely related with hunting pressure [41], [42]. Our results indicate that other long-lived species, which are likely to accumulate lead in their organs, could be at risk of contamination. Many studies in numerous countries have identified lead poisoning as a major risk for wild populations including species of conservation concern like bald and golden eagles [2], [26], [43]. Since 1997, lead shot ammunition for waterfowl hunting was banned in Canada [17]. Therefore, the main source of lead contamination remaining for scavengers is most likely the ingestion of lead embedded in tissues of large ungulate carcasses shot by hunters.

Finally, our results indicate that scavenging on gut piles left by hunters could also contaminate less obvious species such as black bears and coyotes. However, only a few studies have examined lead-contamination in bears. In a recent study conducted in the Greater Yellowstone Ecosystem, Rogers et al. [44] found that black and grizzly bears had higher blood lead concentrations than other carnivores but the authors could not determine the source of the lead intake by the bears. Black bears are opportunistic scavengers [45] that can temporarily deter other scavengers from a carcass and our results showed that they can forage frequently at moose gut piles. In Canada, however, an understanding of the potential sources lead contamination in black bears is still lacking.

Consumption of wild venison shot with lead ammunition can constitute a threat for human health, especially for hunters and autochthonous populations that rely on such resources [1], [46]. Humans are highly vulnerable to lead contamination: a blood lead concentration of 200 µg.L^−1^ is associated with increased mortality rates [47]. Lower concentrations can also induce several physiological disorders [48]. In northern Quebec, lead from ammunition was an important source of lead exposure in Inuit communities before its use was regulated in 1997 and 1999 [49]. However a survey conducted in 2009 showed that this means of lead exposure was still prominent in Inuit populations due to the on-going use of lead ammunition by hunters [20]. In Norway, where 95% of hunters use lead-based ammunition, Lindboe et al. [50] simulated lead intake from moose meat consumption and found that the intake of meat from big game shot with lead ammunition significantly contributed to the total lead exposure in humans.

## Conclusion

Because of the lifetime cumulative nature of sub-lethal exposures, many studies (see above) have already identified the potentially important source of contamination from lead ammunition for both wildlife and human health. However, this type of ammunition is still widely used in most countries [43], [51]. After first banning the use of lead ammunition within the range of the California condor, California has recently decided to extend this regulation to the whole state. Ammunition regulation can indeed benefit to scavengers and predators. The use of non-lead rifle ammunition significantly reduced lead exposure in golden and bald eagles and turkey vulture (*Cathartes aura*) [26], [43]. Because alternative ammunitions exist, such as copper bullets [25], [52] or centerfire bullets designed to resist fragmentation [53], such policy decisions should not be too controversial [43], [54]. Indeed, such alternative ammunition proved to be as efficient as lead ammunition, are generally affordable for hunters and more ethical than lead ones [55]. We hope that this contribution will emphasize the risk posed by lead ammunition to government and policy makers, and we expect that this will encourage regulation review, at least in areas characterised by high big game hunting harvest.
